# Supplementary material for: From sequence to enzyme mechanism using multi-label machine learning
Source: BMC Bioinformatics. 2014 May 19;15:150. doi: 10.1186/1471-2105-15-150 (PMC4229970; doi:10.1186/1471-2105-15-150)
Supplement: Additional file 2 — Java code of ml2db. Additional file ml2db_code.tar.gz contains the Java source code to run the multi-label machine learning experiments and save the results to database. The code’s Javadoc is included. [file 1471-2105-15-150-S2.zip › additional file 2/ml2db/utils/doc/uk/ac/standrews/utils/test/XmlNodeTest.html]

XmlNodeTest


JavaScript is disabled on your browser.


- Overview
- Package
- Class
- Use
- Tree
- Deprecated
- Index
- Help

- Prev Class
- Next Class

- Frames
- No Frames

- All Classes

- Summary:
- Nested |
- Field |
- Constr |
- Method

- Detail:
- Field |
- Constr |
- Method


uk.ac.standrews.utils.test

## Class XmlNodeTest

- java.lang.Object
- - junit.framework.Assert
  - - junit.framework.TestCase
    - - uk.ac.standrews.utils.test.XmlNodeTest

- All Implemented Interfaces:
  :   junit.framework.Test

  ---

    

  ```
  public class XmlNodeTest
  extends junit.framework.TestCase
  ```

  Class

  Version:
  :   18 Nov 2008

  Author:
  :   Luna De Ferrari luna.deferrari-at-ed.ac.uk

- - ### Constructor Summary

    Constructors

    Constructor and Description

    `XmlNodeTest()`
  - ### Method Summary

    Methods

    | Modifier and Type | Method and Description |
    | --- | --- |
    | `static XmlNode` | `getNumberedXmlTree()` |
    | `static XmlNode` | `getPdbSimpleElement()` |
    | `static java.lang.String` | `getXml1String()` |
    | `static java.lang.String` | `getXml2String()` |
    | `static java.lang.String` | `getXml3String()` |
    | `static java.lang.String` | `getXml4String()` |
    | `static java.lang.String` | `getXml5String()` |
    | `static java.lang.String` | `getXml6String()` |
    | `static java.lang.String` | `getXml7String()` |
    | `static java.lang.String` | `getXmlString()` |
    | `void` | `setUp()` |
    | `void` | `testAddChildElement()` |
    | `void` | `testNodeHeadToString()` |
    | `void` | `testSimpleElement()` |
    | `void` | `testToString()` |

    - ### Methods inherited from class junit.framework.TestCase

      `countTestCases, getName, run, run, runBare, setName, toString`
    - ### Methods inherited from class junit.framework.Assert

      `assertEquals, assertEquals, assertEquals, assertEquals, assertEquals, assertEquals, assertEquals, assertEquals, assertEquals, assertEquals, assertEquals, assertEquals, assertEquals, assertEquals, assertEquals, assertEquals, assertEquals, assertEquals, assertEquals, assertEquals, assertFalse, assertFalse, assertNotNull, assertNotNull, assertNotSame, assertNotSame, assertNull, assertNull, assertSame, assertSame, assertTrue, assertTrue, fail, fail, failNotEquals, failNotSame, failSame, format`
    - ### Methods inherited from class java.lang.Object

      `equals, getClass, hashCode, notify, notifyAll, wait, wait, wait`

- - ### Constructor Detail


    - #### XmlNodeTest

      ```
      public XmlNodeTest()
      ```
  - ### Method Detail


    - #### getNumberedXmlTree

      ```
      public static XmlNode getNumberedXmlTree()
      ```


    - #### getPdbSimpleElement

      ```
      public static XmlNode getPdbSimpleElement()
      ```

      Returns:
      :   an xml node containing PDB data


    - #### getXml1String

      ```
      public static java.lang.String getXml1String()
      ```


    - #### getXml2String

      ```
      public static java.lang.String getXml2String()
      ```


    - #### getXml3String

      ```
      public static java.lang.String getXml3String()
      ```


    - #### getXml4String

      ```
      public static java.lang.String getXml4String()
      ```


    - #### getXml5String

      ```
      public static java.lang.String getXml5String()
      ```


    - #### getXml6String

      ```
      public static java.lang.String getXml6String()
      ```


    - #### getXml7String

      ```
      public static java.lang.String getXml7String()
      ```


    - #### getXmlString

      ```
      public static java.lang.String getXmlString()
      ```


    - #### setUp

      ```
      public void setUp()
                 throws java.lang.Exception
      ```

      **Overrides:**
      :   `setUp` in class `junit.framework.TestCase`

      Throws:
      :   `java.lang.Exception`


    - #### testAddChildElement

      ```
      public void testAddChildElement()
      ```


    - #### testNodeHeadToString

      ```
      public void testNodeHeadToString()
      ```


    - #### testSimpleElement

      ```
      public void testSimpleElement()
      ```


    - #### testToString

      ```
      public void testToString()
      ```


- Overview
- Package
- Class
- Use
- Tree
- Deprecated
- Index
- Help

- Prev Class
- Next Class

- Frames
- No Frames

- All Classes

- Summary:
- Nested |
- Field |
- Constr |
- Method

- Detail:
- Field |
- Constr |
- Method
